# Meal planning is associated with food variety, diet quality and body weight status in a large sample of French adults

**DOI:** 10.1186/s12966-017-0461-7

**Published:** 2017-02-02

**Authors:** Pauline Ducrot, Caroline Méjean, Vani Aroumougame, Gladys Ibanez, Benjamin Allès, Emmanuelle Kesse-Guyot, Serge Hercberg, Sandrine Péneau

**Affiliations:** 1 Equipe de Recherche en Epidémiologie Nutritionnelle, Centre de Recherche en Epidémiologie et Statistiques, Université Paris 13, Inserm (U1153), Inra (U1125), Cnam, COMUE Sorbonne Paris Cité, Bobigny, France; 2 Département de médecine générale, faculté de médecine Pierre et Marie Curie, UPMC Université Paris 6, 27, rue de Chaligny, 75012 Paris, France; 3 Département de Santé Publique, Hôpital Avicenne, Bobigny Cedex, France

**Keywords:** Meal planning, Diet quality, Food variety, Overweight, Cross-sectional study

## Abstract

**Background:**

Meal planning could be a potential tool to offset time scarcity and therefore encourage home meal preparation, which has been linked with an improved diet quality. However, to date, meal planning has received little attention in the scientific literature. The aim of our cross-sectional study was to investigate the association between meal planning and diet quality, including adherence to nutritional guidelines and food variety, as well as weight status.

**Methods:**

Meal planning, i.e. planning ahead the foods that will be eaten for the next few days, was assessed in 40,554 participants of the web-based observational NutriNet-Santé study. Dietary measurements included intakes of energy, nutrients, food groups, and adherence to the French nutritional guidelines (mPNNS-GS) estimated through repeated 24-h dietary records. A food variety score was also calculated using Food Frequency Questionnaire. Weight and height were self-reported. Association between meal planning and dietary intakes were assessed using ANCOVAs, while associations with quartiles of mPNNS-GS scores, quartiles of food variety score and weight status categories (overweight, obesity) were evaluated using logistic regression models.

**Results:**

A total of 57% of the participants declared to plan meals at least occasionally. Meal planners were more likely to have a higher mPNNS-GS (OR quartile 4 *vs*. 1 = 1.13, 95% CI: [1.07–1.20]), higher overall food variety (OR quartile 4 *vs*. 1 = 1.25, 95% CI: [1.18–1.32]). In women, meal planning was associated with lower odds of being overweight (OR = 0.92 [0.87–0.98]) and obese (OR = 0.79 [0.73–0.86]). In men, the association was significant for obesity only (OR = 0.81 [0.69–0.94]).

**Conclusions:**

Meal planning was associated with a healthier diet and less obesity. Although no causality can be inferred from the reported associations, these data suggest that meal planning could potentially be relevant for obesity prevention.

**Electronic supplementary material:**

The online version of this article (doi:10.1186/s12966-017-0461-7) contains supplementary material, which is available to authorized users.

## Background

In industrialized countries, eating habits and cooking practices have considerably changed. First, time devoted to cooking has decreased: in the United States, it has been reduced from 1:63 hour per day in 1965–1966 to 58 min in 2006–2007 [1]. Additionally, the source of food consumed has changed: people consume less food prepared at home, whereas foods prepared away from home represent an increasing part of the diet [2–4].

In light of this observation, a number of studies have evaluated the potential impact of food prepared away from home on dietary quality, as well as weight status. These studies highlighted that the consumption of food prepared away from home is associated with a lower quality diet [5–8] and a higher body mass index [9–11], whereas benefits have been attributed to home-prepared food [2, 12–14]. More frequent home food preparation has been associated with better adherence to dietary objectives [12], higher intakes of fruits, vegetables [13, 14], fiber, folate and vitamin A, while lower intakes of fat in young people [13]. Therefore, home meal preparation has been increasingly promoted as a strategy for improving dietary quality and preventing obesity [12–15].

In designing strategies to promote home cooking, it is important to understand the patterns and correlates of home meal practices. Many studies have investigated the reasons why people cook less. Time scarcity and cooking skills were identified as common barriers to prepare home meals [6, 11, 12, 16, 17]. Previous research emphasized that individuals with lower cooking skills were more likely to consume away from home food such as ready meals or take-out meals from fast food or restaurants [11, 18]. In response to these difficulties, a number of studies have evaluated the opportunity to improve cooking skills in order to promote healthy dietary patterns [19–21]. To face time pressure, a series of qualitative studies highlighted that parents resort to food choice coping strategies, such as meal simplification, taking out, or meal planning [16, 17, 22–26] despite their potential impact on diet quality. Among these strategies, time management skills [27] and in particular meal planning [28, 29], which consists in deciding ahead the foods that will be eaten in the next few days, has been previously suggested as a solution to balance competing time demands and reduce barriers to healthy dietary practices. In the literature, very few studies have investigated meal planning practices and they often focused on adequate diet for diabetic subjects [30–32]. Studies performed on general populations showed that meal planning was positively associated with frequencies of home food preparation [29] and family meal [33], as well as the presence of fruits for dinner [34]. To our knowledge, only one study in the literature has evaluated the potential link between meal planning and food consumption. It focused on fruit and vegetables specifically, and showed that planning meal ahead was associated with higher fruit and vegetable intakes [35]. However, the latter presented weakness in the dietary intake assessment method since it consisted only of questions on the number of servings eaten per day. Additionally, meal planning was evaluated, among various practices, as a tool to maintain weight among successful weight losers [36, 37] but no data exists on the potential relationship with weight status in the general population. In the present study, we hypothesize that meal planning might encourage home meal preparation, and therefore have beneficial effects on dietary quality and consequently on weight status. Thus, we first described meal planning practices among a large sample of individuals. Then, we investigated the relationships between meal planning and diet quality, based on adherence to nutritional guidelines, energy, macronutrients and food group intakes, as well as food variety. Finally, we evaluated the association between meal planning and weight status.

## Methods

### Study population

The NutriNet-Santé study (http://info.etude-nutrinet-sante.fr) is an ongoing web-based prospective observational cohort study launched in France in May 2009 with a scheduled follow-up of 10 years. It aims to investigate the relationship between nutrition and chronic disease risk, as well as the determinants of dietary behavior and nutritional status. The study was implemented in the French general population (internet-using adult volunteers, aged ≥18 years). The rationale, design and methodology of the study have been fully described elsewhere [38]. In brief, to be included into the study, participants have to complete a baseline set of self-administered web-based questionnaires assessing dietary intake, physical activity, anthropometric characteristics, lifestyle, socioeconomic conditions and health status. As part of the follow-up, participants are asked to complete the same set of questionnaires each year. Moreover, each month, participants are invited by e-mail to fill in optional questionnaires related to dietary intake, determinants of eating behaviors, nutritional and health status. This study is conducted in accordance with the Declaration of Helsinki, and all procedures were approved by the Institutional Review Board of the French Institute for Health and Medical Research (IRB Inserm n°0000388FWA00005831) and the *Commission Nationale de l’Informatique et des Libertés* (CNIL n°908450 and n°909216). All participants provided informed consent with an electronic signature. This study is registered in EudraCT (n°2013-000929-31).

### Data collection

#### Meal planning questionnaire

Meal planning practices were assessed via an optional questionnaire launched in the NutriNet-Santé cohort study in April 2014.

First, grocery shopping and cooking practices were evaluated. In particular, participants were asked to indicate whether they were involved in grocery shopping (every day, several times a week, once a week, less than once a week) and cooking (every day twice a day, every day once a day, several times a week but not every day, once a week, less than once a week, never) in their household. Then, participants were asked the following question “Generally, when do you choose the foods you are going to eat for meal?” (just before meal, during the day, the day before, few days before, one week before, never). Participants responding “never” were exempted to complete the rest of the questionnaire.

Participants were also asked whether having to think about what they have to cook is a constraint for them. The responses were rated on a 5-point Likert scale ranging from one (strongly disagree) to five (strongly agree).

Participants were then asked whether they planned meals, even in an irregular manner (yes I do, yes I did but not anymore, no I never planned meals). The definition of “planning meals” given to the participants was “to plan ahead the foods that will be eaten for the next few days”. Participants who reported planning meal currently were considered as “meal planners” whereas others were categorized as “non-meal planners”.

Finally, the questionnaire included questions about meal planning frequency (several times a week, once a week, once every two weeks, two to three times a month, not regularly), duration (a few days, one week, two weeks or more), period of the week (weekdays, weekend, weekdays and weekend) and sources of inspiration (personal recipe repertoire, Internet or apps, ingredients available during grocery shopping).

#### Socio-demographic and economic characteristics

At baseline and annually thereafter, participants in the NutriNet-Santé study are asked to provide socio-demographic data, including sex, age (18–30, 30–50, 50–65, >65 years), educational level (up to secondary, some college or university degree), monthly income (<1,200 €, 1,200–1,800 €, 1,800–2,700 € and >2,700 € per consumption unit), presence of children in the household (yes, no), history of dieting to lose weight during the past year (yes, no) and physical activity (low, moderate, high). Monthly household income is calculated per “consumption unit” (CU), where one CU is attributed for the first adult in the household, 0.5 CU for other persons aged 14 or older, and 0.3 CU for children under 14, following national statistics methodology and guidelines [39].

Physical activity was assessed using a short form of the French version of the International Physical Activity Questionnaire (IPAQ). The weekly energy expenditure expressed in metabolic equivalent task minutes per week was estimated, and three scores of physical activity were constituted [i.e., low (<30 min/day), moderate (30–59 min/day), and high (≥60 min/day)] according to the French guidelines for physical activity [40].

For the present study, we used the closest available data with respect to the assessment of meal planning practices.

#### Dietary measurements

At inclusion and once a year thereafter, participants are invited to complete three non-consecutive 24-h dietary records, randomly assigned over a 2-week period (two weekdays and one weekend day). For the present analysis, we selected participants who completed at least three 24-h dietary records since their inclusion in the cohort study (i.e. completed between May 2009 and December 2014). Participants reported all foods and beverages consumed at each eating occasion. They estimated the amounts eaten using validated photographs of portion sizes [41], using household measures or by indicating the exact quantity (grams) or volume (milliliters). Daily mean food intakes were calculated, weighted for the type of day of the week. Energy, nutrient and food group intakes were estimated using the NutriNet-Santé composition table including more than 2000 foods [42]. Dietary underreporting was identified on the basis of the method proposed by Black [43]. We hypothesize that meal planning encourages food preparation and therefore considered food groups that can be used in food preparation (e.g. eggs). In addition, we considered food groups that have nutritional interest (e.g. fruits). Thus, the following food groups were included in the study: fruits, vegetables, fish (including seafood and processed seafood), meat (including cooked ham, offal), eggs, milk, cheese, added fats (including oil, butter, margarine, vinaigrette), sugary products (e.g. cake, biscuits, sugars, honey, jam, chocolate) and starchy foods (including potato, legumes, pasta, rice, other cereals) with a specific focus on legumes and whole grain starchy foods (including whole grain pasta, rice, other cereals).

Adherence to nutritional guidelines was assessed using the PNNS Guideline Score (PNNS-GS). The 15-point PNNS-GS is a validated a priori score reflecting the adherence to the official French nutritional guidelines which has been extensively described elsewhere [44]. Details on computation of this score are in Additional file 1. Briefly, it includes 13 components: eight refer to food-serving recommendations (fruit and vegetables; starchy foods; whole grain products; dairy products; meat, eggs and fish; fish and seafood; vegetable fat; water *vs*. soda), four refer to moderation in consumption (added fat; salt; sweets; alcohol) and one component pertains to physical activity [44, 45]. Points are deducted for overconsumption of salt (>12 g/day), added sugars (>17.5% of energy intake), or when energy intake exceeds the needed energy level by more than 5%. Each component cut-off was that of the threshold defined by the PNNS public health objectives when available [45] otherwise they were established according to the French Recommended Dietary Allowances [46]. For the present analysis, we consider the mPNNS-GS, a modified version of the PNNS-GS, which takes into account only the dietary components, therefore excluding the physical activity component. Thus, the maximum score was 13.5.

#### Food variety score

Food variety has been defined as the number of different food items reported to be eaten over a given reference period [47]. Considering that seasonality is likely to influence food variety and that a period of 10 to 15 days has been recommended to accurately assess food variety [48], the food variety was evaluated using a Food Frequency Questionnaire (FFQ).

Sixteen months after baseline, participants were invited to complete a self-administrated 240-items FFQ to assess their usual dietary intake over the past year [49]. Participants were asked to report their consumption frequency on the basis of how many times they ate the standard portion size proposed (typical household measurements such as spoon or standard unit such as a yogurt). The frequency of consumption referred to usual consumption over the past year on an increasing scale including yearly, monthly, weekly or daily units, as suitable, and participants were asked to provide only one answer.

The food variety score corresponded to the number of FFQ items reported to be consumed at least once during the last year [47]. The maximum score was therefore 240. Fruit and vegetable variety scores were also computed based on the number of different fruits and vegetables reported by the participants.

#### Anthropometric data

Height and weight were assessed by using an anthropometric questionnaire, which was self-administered online, at baseline and each year thereafter [50, 51]. For each participant, the closest available data to the meal planning questionnaire were used for the analysis.

Data were not collected for pregnant women. BMI (in kg/m^2^) was calculated as the ratio of weight to squared height. Participants were classified as underweight or normal weight (BMI < 25), overweight (25 ≤ BMI < 30) and obesity (BMI ≥ 30) according to WHO references values [52].

### Statistical analysis

The analysis focused on participants who had completed the meal planning questionnaire, had declared being involved in meal preparation in their household, and who had completed at least three 24-h dietary records since they were included in the study, as well as the FFQ.

Chi-square tests and Student’s *t* tests were used to compare characteristics of included *vs*. excluded participants, as well as meal planners *vs*. non-meal planners. Meal planners’ practices were also described. Continuous variables are presented as means ± SDs and categorical variables as percentages.

ANCOVAs were performed to investigate the relationship between meal planning and energy, macronutrients and food groups. However, for some particular food groups which did not exhibit normal distribution (i.e. eggs, milk, legumes, and whole grain starchy foods), mainly due to a high proportion of non-consumers, a binary variable (consumer/non-consumer) was created and a logistic regression analysis was performed. Logistic regression models were also used to assess the associations between meal planning and quartiles of mPNNS-GS, as well as quartiles of food variety scores (overall, fruit and vegetable) and BMI categories. Due to significant interactions and differences on the associations with meal planning, analyses on BMI were performed separately by sex.

Meal planning has been described as a cooking skill [53]. Thus, characteristics that have been shown to influence cooking practices, dietary intakes or weight status were considered as confounders in the present analyses. Models were therefore all adjusted for sex [1, 54, 55], age [56], educational level, monthly income [6], presence of children in the household [6], history of dieting to lose weight during the past year [57], physical activity [58], and cooking frequency. Models evaluating the associations with mPNNS-GS, macronutrient and food groups intakes were further adjusted for daily energy intake and number of 24-h dietary records completed by participants. The energy model was only adjusted on the number of 24-h records while the food variety models were adjusted on daily energy intake. Missing covariate data were imputed using multiple imputation method.

Sensitivity analyses were conducted on a subsample of individuals having responded to at least one of the dietary assessments (i.e. FFQ, dietary records). In addition, analyses were conducted using another definition of food variety score (number of FFQ items reported to be consumed more than once a week) [59].

All tests of statistical significance were two-sided and the type I error was set at 5%. Statistical analyses were performed using SAS software (version 9.3, SAS Institute Inc, Cary, NC, USA).

## Results

Among the 102,703 participants in the NutriNet-Santé study who received the meal planning questionnaire, a total of 52,949 participants (i.e. 51.6%) completed it. Among them, 1,754 were excluded because they declared not being involved in meal preparation in their household, 3,242 because of inadequate data in dietary records (less than three 24-h dietary records or underreporting) and 7,399 because they did not complete the FFQ, thus leading to a total of 40,554 participants available for analyses. Compared with excluded participants, included subjects were more likely to be women, older, to have a lower educational level, higher income, to have children living in the household, to be physically active, and less likely to have followed a diet to lose weight during the past year (all *P* < 0.0001).

Our final sample comprised 78% of women and 22% of men, with a mean age of 52.2 ± 14.2 years. Among the included participants, 57.4% declared to plan their meals at least occasionally whereas 42.6% did not, among which 17.3% planned in the past and 25.3% never planned meals. Overall, the same proportions were observed in men (meal planners: 55.9% *vs*. non-meal planners: 44.1%) and women (meal planners: 57.8% *vs*. non-meal planners: 42.2%), but women were more likely to have planned meals in the past compared to men (19.0% *vs*. 11.3%).

Table 1 presents the sociodemographic and economic characteristics of meal planners and non-meal planners, as well as mean scores for mPNNS-GS, overall food variety and overweight prevalence. Overall, differences between the two groups were relatively limited. Compared with non-meal planners, individuals who plan meals were slightly more likely to be women, older, to have a higher educational level, a higher income, to have followed a diet to lose weight during the past year and to be physically active (all *P* < 0.05). They were also more likely to have higher mPNNS-GS and overall food variety scores and to have a BMI < 25 kg/m^2^ (all *P* < 0.0001).

**Table 1:** Sociodemographic, economic and lifestyle characteristics of meal planners *vs*. non-meal planners (*N* = 40,554 - NutriNet-Santé 2014)

	Non-meal planners	Meal planners^a^	
	*N* = 17,271	*N* = 23,283	
	% or means ± SD	% or means ± SD	*P* ^b^
Sex			***0.0011***
Men	22.45	21.09	
Women	77.55	78.91	
Age			***0.031***
18-30	7.24	6.80	
30-50	33.56	33.66	
50-65	35.80	35.07	
> 65	23.40	24.46	
Educational level			***<0.0001***
Up to secondary	34.79	31.84	
Some college	30.85	31.23	
University degree	34.18	36.79	
Missing data	0.18	0.15	
Monthly income per household (€/UC^c^)			***<0.0001***
<1,200	11.50	9.17	
1,200–1,800	22.22	20.48	
1,800–2,700	25.18	25.10	
>2,700	26.63	31.09	
Missing data	14.48	14.16	
Presence of child in the household			0.76
No	26.84	26.97	
Yes	73.16	73.03	
History of dieting to lose weight during the past year			***0.0003***
No	68.52	66.79	
Yes	29.95	31.36	
Missing data	1.53	1.84	
Physical activity level			***<0.0001***
High	30.13	32.32	
Intermediate	35.52	37.15	
Low	21.26	19.32	
Missing data	13.09	11.21	
mPNNS-GS^d^	7.84 ± 1.35	7.95 ± 1.34	***<0.0001***
Food variety score^e^	141.43 ± 27.13	144.49 ± 26.01	***<0.0001***
BMI			***<0.0001***
<25	64.85	68.15	
[25–30]	24.27	23.33	
≥30	10.88	8.53	

Boldface indicates statistical significance

^a^Meal planners are individuals who “plan ahead the foods that will be eaten for the next few days”

^b^On the basis of Student’s t or chi-square tests as appropriate

^c^ *CU*: Household Consumer Units. One CU is attributed for the first adult in the household, 0.5 for other persons aged 14 or older and 0.3 for children under 14

^d^mPNNS-GS: adherence to nutritional guidelines score, based on 24-h dietary records, range 0–13.5

^e^Food variety score: based on the food frequency questionnaire, range 0–240

Table 2 shows cooking practices in meal planners *vs*. non-meal planners, as well as details regarding meal practices among meal planners. Compared with non-meal planners, individuals who plan meals cooked more frequently. The majority of non-meal planners decided what food to prepare during the day or just before meal whereas meal planners reported to decide during the day, the day before or few days before. Finally, thinking about what food to prepare was less of a constraint for meal planners than for non-meal planners (all *P* < 0.0001). Results among meal planners more specifically showed that the majority of participants planned their meals at least once a week. A non-negligible part (14.8%) also reported to plan meals not regularly. Three-quarters of participants planned meals for a few days, but less than a week. Meals were mostly planned for both weekdays and weekend. Most of the participants planned meals according to personal recipe repertoire or the ingredients available during grocery shopping.

**Table 2:** Cooking practices and meal planning practices (*N* = 40,554 - NutriNet-Santé 2014)

	Non-meal planners	Meal planners	
	*N* = 17,271	*N* = 23,283	
	%	%	*P* ^a^
Cooking frequency			***<0.0001***
Every day, twice a day or more	28.79	33.60	
Every day, once a day	34.70	37.06	
Several times a week	27.87	25.30	
Once a week or less	7.14	3.54	
Never	1.51	0.50	
Time of meal choice decision			***<0.0001***
One week before	0.32	7.69	
Few days before	6.02	28.54	
The day before	21.46	25.63	
During the day	41.72	26.27	
Just before meal	30.48	11.87	
Having to think about what to cook is a constraint			***<0.0001***
Strongly agree	9.45	4.33	
Agree	29.52	23.62	
Neither agree nor disagree	27.40	27.04	
Disagree	18.69	23.46	
Strongly disagree	14.94	21.54	
Meal planning frequency			
Several times a week		46.39	
Once a week		34.72	
Two weeks per month or less		4.07	
Not regularly		14.81	
Meal planning duration			
Two weeks or more		1.18	
One week		19.77	
A few days		79.05	
Meal planning period			
Weekdays and weekend		68.15	
Weekdays		22.83	
Weekend		9.02	
Sources of inspiration			
Personal recipe repertoire		41.15	
Internet, apps for meal planning		2.53	
Ingredients available during grocery shopping		56.32	

Boldface indicates statistical significance

^a^On the basis of chi-square tests

Intake of energy, nutrients and food groups in meal planners *vs*. non-meal planners are presented in Table 3. Depending on the outcome, the percentage of explained variance (r^2^) in ANOVAs varied from 0.10 to 0.75. Overall very small differences in energy, macronutrient and food group intakes were observed between meal planners and non-meal planners.

**Table 3:** Energy, nutrients and food group intakes in meal planners *vs*. meal planners (*N* = 40,554 - NutriNet-Santé 2014)

	Univariable			Multivariable		
	Non-meal planners	Meal planners		Non-meal planners	Meal planners	
	*N* = 17,271	*N* = 23,283		*N* = 17,271	*N* = 23,283	
**Energy**	**Mean ± SE**	**Mean ± SE**	***P***	**Mean ± SE**	**Mean ± SE**	***P*** ^1^
Energy (kcal/d)	1867.11 ± 3.22	1865.01 ± 2.77	0.62	1869.78 ± 2.78	1863.03 ± 2.39	0.068
**Nutrients**	**Mean ± SE**	**Mean ± SE**	***P***	**Mean ± SE**	**Mean ± SE**	***P*** ^2^
Lipids (g/d)	80.23 ± 0.17	80.15 ± 0.14	0.69	80.21 ± 0.08	80.17 ± 0.07	0.76
Saturated fatty acids (g/d)	32.76 ± 0.08	32.9 ± 0.07	0.17	32.72 ± 0.05	32.93 ± 0.04	***0.001***
Proteins (g/d)	77.3 ± 0.14	77.69 ± 0.12	***0.038***	77.22 ± 0.09	77.74 ± 0.08	***<0.0001***
Carbohydrates (g/d)	193.23 ± 0.39	191.98 ± 0.34	***0.015***	193.16 ± 0.22	192.04 ± 0.19	***0.0001***
Sugars (g/d)	90.21 ± 0.22	90.63 ± 0.19	0.14	90.27 ± 0.17	90.58 ± 0.14	0.17
**Food groups**	**Mean ± SE**	**Mean ± SE**	***P***	**Mean ± SE**	**Mean ± SE**	***P*** ^2^
Fruits (g/d)	195.31 ± 0.97	202.15 ± 0.83	***<0.0001***	197.43 ± 0.92	200.58 ± 0.79	***0.011***
Vegetables (g/d)	303.78 ± 1.11	317.76 ± 0.96	***<0.0001***	307.68 ± 1.05	314.86 ± 0.91	***<0.0001***
Fish (g/d)	69.39 ± 0.35	71.24 ± 0.3	***<0.0001***	70.3 ± 0.33	70.57 ± 0.29	0.56
Meat (g/d)	116.87 ± 0.44	118.7 ± 0.38	***0.0016***	117.29 ± 0.41	118.4 ± 0.35	***0.040***
Cheese (g/d)	35.52 ± 0.17	35.8 ± 0.15	0.22	35.53 ± 0.16	35.8 ± 0.13	0.18
Starchy foods (g/d)^a^	227.81 ± 0.83	225.56 ± 0.71	***0.040***	230.41 ± 0.73	223.63 ± 0.63	***<0.0001***
Added fats (g/d)	47.17 ± 0.16	48.03 ± 0.14	***<0.0001***	47.35 ± 0.14	47.89 ± 0.12	***0.0032***
Sugary products (g/d)^b^	145.71 ± 0.57	146.01 ± 0.49	0.69	146.6 ± 0.48	145.34 ± 0.42	0.050
	**Ref**	**OR[95% CI]**	***P***	**Ref**	**OR[95% CI]**	***P*** ^3^
Eggs	1	1.17 [1.10;1.23]	***<0.0001***	1	1.00 [0.94;1.06]	0.93
Milk	1	1.11 [1.06;1.17]	***<0.0001***	1	0.97 [0.92;1.01]	0.12
Legumes	1	1.09 [1.04;1.13]	***<0.0001***	1	0.96 [0.92;1.00]	0.070
Whole grain starchy foods^c^	1	1.04 [1.00;1.09]	0.062	1	1.04 [0.99;1.09]	0.14

Boldface indicates statistical significance

^1^ *P* are based on ANCOVA models adjusted for sex, age, educational level, monthly income per household, presence of children in the household, history of dieting to lose weight during the past year, physical activity, cooking frequency, and number of dietary records

^2^ *P* are based on ANCOVA models adjusted for sex, age, educational level, monthly income per household, presence of children in the household, history of dieting to lose weight during the past year, physical activity, cooking frequency, number of dietary records, and daily energy intake

^3^ *P* are based on logistic regression models adjusted for sex, age, educational level, monthly income per household, presence of children in the household, history of dieting to lose weight during the past year, physical activity, cooking frequency, number of dietary records, and daily energy intake

^a^Total starchy foods includes potato, legumes, pasta, rice, other cereals, flour and whole grain forms

^b^Sugary products includes foods with high sugar content such as cake, biscuits, sugars, honey, jam, chocolate

^c^Whole grain starchy foods includes whole grain forms of pasta, rice, other cereals, and flour

The associations between meal planning and quartiles of mPNNS-GS, as well as quartiles of food variety score are presented in Table 4.

**Table 4:** Multinomial logistic regression analysis showing the association between meal planning and adherence to nutritional guideline score (mPNNS-GS) and food variety score (*N* = 40,554 - NutriNet-Santé 2014)^a^

	Univariable		Multivariable	
	OR [95% CI]	*P*	OR [95% CI]	*P* ^b^
Adherence to nutritional guidelines (mPNNS-GS score)				
Q1 (<6.91)	1		1	
Q2 ([6.91–7.83])	1.10 [1.04;1.17]	0.0006	1.06 [1.00;1.13]	***0.039***
Q3 ([7.83–8.8])	1.16 [1.10;1.23]	<0.0001	1.10 [1.03;1.16]	0.0022
Q4 (≥8.8)	1.23 [1.16;1.30]	<0.0001	1.13 [1.07;1.20]	<0.0001
Food variety score				
Overall				
Q1 (<127)	1		1	
Q2 ([127–146])	1.15 [1.09;1.22]	<0.0001	1.15 [1.08;1.22]	<0.0001
Q3 ([146–162])	1.28 [1.21;1.36]	<0.0001	1.16 [1.10;1.23]	<0.0001
Q4 (≥162)	1.34 [1.27;1.42]	<0.0001	1.25 [1.18;1.32]	<0.0001
Vegetables				
Q1 (<20)	1		1	
Q2 ([20–23])	1.22 [1.15;1.29]	<0.0001	1.18 [1.11;1.25]	<0.0001
Q3 ([23–25])	1.33 [1.26;1.41]	<0.0001	1.24 [1.17;1.32]	<0.0001
Q4 (≥25)	1.38 [1.3;1.46]	<0.0001	1.21 [1.14;1.28]	<0.0001
Fruits				
Q1 (<15)	1		1	
Q2 ([15–17])	1.13 [1.06;1.20]	<0.0001	1.07 [1.01;1.13]	0.032
Q3 ([17–19])	1.21 [1.14;1.28]	<0.0001	1.12 [1.06;1.19]	0.0002
Q4 (≥19)	1.23 [1.17;1.31]	<0.0001	1.12 [1.06;1.19]	<0.0001

Boldface indicates statistical significance

^a^The modeled probability was the fact to plan meals

^b^Adjusted for sex, age, educational level, monthly income per household, presence of children in the household, history of dieting to lose weight during the past year, physical activity cooking frequency, and daily energy intake

Compared with non-meal planners, individuals who planned their meals were more likely to belong to quartiles 2, 3 and 4 of mPNNS-GS compared with quartile 1, thus reflecting a higher adherence to nutritional guidelines. Similarly, compared with non-meal planners, meal planners were also more likely to belong to quartiles 2, 3 and 4 of overall food variety, vegetable variety and fruit variety compared with quartile 1, thus reflecting a higher variety of the diet. For these models, the association of predicted probabilities and observed responses indicated percent concordant of 63.8 and 54.9%, respectively*.* Additional analysis considering mPNNS-GS and variety score as continuous variables revealed similar trends: meal planners exhibited higher mPNNS-GS (7.92 ± 0.008 *vs*. 7.88 ± 0.009, *P* = 0.0001) and overall food variety score (113.81 ± 0.16 *vs*. 112.20 ± 0.19, *P* < 0.0001) compared to non-meal planners.

The logistic regression analysis performed between meal planning and BMI classes is presented in Table 5. In women, meal planning was associated with lower odds of being overweight and obese, while in men, meal planning was associated with lower odds of being obese only. For this model, the association of predicted probabilities and observed responses indicated a percent concordant of 70%.

**Table 5:** Logistic regression analysis showing the association between meal planning and weight status in men and women (*N* = 40,554 - NutriNet-Santé 2014)^a^

	Men (*N* = 8,788)				Women (*N* = 31,766)			
	Univariable		Multivariable		Univariable		Multivariable	
	OR [95% CI]	*P*	OR [95% CI]	*P* ^b^	OR [95% CI]	*P*	OR [95% CI]	*P* ^b^
BMI<25	1		1		1		1	
[25–30]	0.98 [0.89;1.07]	0.60	1.00 [0.90;1.10]	0.93	0.91 [0.86;0.96]	0.0005	0.92 [0.87;0.98]	0.0081
≥30	0.78 [0.68;0.90]	0.0008	0.81 [0.69;0.94]	0.0065	0.74 [0.69;0.80]	<0.0001	0.79 [0.73;0.86]	<0.0001

^a^The modeled probability was the fact to plan meals

^b^Adjusted for sex, age, educational level, monthly income per household, presence of children in the household, history of dieting to lose weight during the past year, physical activity, and cooking frequency

## Discussion

Using a large population-based sample of individuals, this study brought new insights about meal planning practices and their relationship with dietary quality and weight status. Meal planning was associated with better adherence to nutritional guidelines and higher food variety. Furthermore, planning meals was associated with lower odds of being overweight and obese in women and of being obese in men.

In our study, despite significant differences regarding sociodemographic, economic and lifestyle characteristics due to the large sample size, meal planners and non-meal planners exhibited very similar profiles. In particular, no significant difference in meal planning was observed in relation with the presence of children in the household. This result appears in contrast with previous qualitative studies suggesting that the presence of children increases the feeling of time scarcity [17, 23–26, 60] and therefore the need of developing time-saving strategies, such as meal planning. However, fatigue and time scarcity can also decrease the likelihood for following meal plans [26].

To our knowledge, this study is the first to describe meal planning practices in a general population sample. Overall, more than one out two participants revealed to plan their meals at least occasionally. Generally, individuals planned their meals several times a week, for a few days period including weekdays and weekend, and get inspiration mostly from their personal recipe repertoire or ingredients available during grocery shopping. A previous survey evaluating Canadians’ attitudes and habits with regard to home food preparation highlighted that about 40% of the participants decide what they will prepare for dinner during the day, 27% the day before and 33% at least two days before [29]. However, the latter did not explore the modalities of meal planning.

Based on a large sample of general population, our data support the notion that planning meal is indeed associated with a better adherence to nutritional guidelines and an increased food variety (overall, fruits and vegetables). However, it should be noted that only small differences were observed with energy, macronutrient and food group intakes specifically. Although meal planning has been previously suggested as a potential tool to improve dietary quality [28, 29], to our knowledge, no study in the literature has investigated this related association. Previous authors highlighted that individuals deciding in advance what to prepare for dinner were more likely to cook homemade dishes [29]. Since more frequent food preparation has been linked with a better diet quality [12–14], this could potentially explain the healthier diet observed in meal planners. A few hypotheses can be made on how meal planning could encourage home food preparation. First, meal planning might address the issue of not knowing what to prepare for dinner, that has been previously described as a barrier for home meal preparation [29]. Second, by planning meals individuals may think about recipes that can be prepared in a limited period of time and, therefore reduce the feeling of time scarcity, that may limit home meal preparation [6, 12, 16] and increase the recourse to food choice coping strategies such as eating out, delivery meals or ready prepared food [17, 23–25]. In addition, planning meals may reduce the risk of missing ingredients for home meal preparation which could also lead to the consumption of food prepared away from home. Finally, deciding what foods will be eaten in the next few days could also enable individuals to cook more diversified recipes and to anticipate grocery shopping for the specific ingredients needed, thus potentially explaining the increased food variety observed in meal planners. However, reverse causality cannot be excluded since individuals interested in having a healthy diet might be more likely to plan their meals. In line with this idea, meal-planners in our sample were more likely to have higher educational level, to have higher income, to be physically active and to have a lower BMI, characteristics that have been related with a better attitude towards healthy eating [61]. Differences were however limited.

Our results showed that women who planned meals were less likely to be overweight or obese, while in men, there was an association with obesity only. Since meal planners have a diet of higher quality, it potentially prevents overweight in these individuals [52]. However, we cannot exclude reverse causality. People attaching more importance to food and weight management might be more likely to plan their meals. In line with this hypothesis, two studies in the literature highlighted that meal planning is more frequently used by successful weight loss maintainers compared to those who did not maintain weight losses [36, 37].

In terms of public health, our results bring supportive insights that promoting meal planning might encourage the preparation of healthier and more varied home meals. Previous studies showed that parents would be interested in learning how to plan meals [28, 60], however, other findings suggested that meal planning is also perceived as complex and time consuming [62]. Specific tools might assist people in managing meal planning but to be adopted and sustainable over time, it is important to identify consumers’ needs. The present data highlighted that there are various ways of planning meals. As an example, we observed that the ingredients available during grocery shopping are likely to influence meal planning while existing tools rather propose menus to plan grocery shopping.

### Strengths and limitations

A major strength of this study was its large sample size allowing an evaluation of meal planning practices at a population level. The wide range of socio-economic and lifestyle variables collected through the web-based platform enables the control of potential effects of confounding factors. In addition, the web-based tool used to assess 24-h dietary records has shown a good validity in prior studies [63, 64]. However, because of the influence of seasonality on food variety, the FFQ was used to evaluate dietary variety since it allows usual intake estimates over a relatively long period of time [65].

This study was also subject to several limitations. First, the cross-sectional design of this study prevented any inference of causality. Moreover, since participants were volunteers in a nutrition focused cohort, they may have higher health consciousness and interest in nutritional issues. The fact that we selected only participants who completed both dietary assessment tools might have exacerbated this characteristic in our sample. Therefore, caution is needed when generalizing our results. However, sensitivity analyses including individuals with at least one of the dietary assessments (24-h dietary records or FFQ) revealed similar trends. Besides, the fact that participants had relatively high knowledge in nutrition could potentially account for the few differences observed in energy and food group intakes, since they may be able to cook healthful meals without planning meals. It is also important to consider that food variety score was based on FFQ data, which has been recorded at different time frames (16 months after the inclusion in the cohort). Thus, for participants included since a long time in the cohort study, the estimation may not represent their current dietary repertoire. In addition, data were self-reported, thus potentially leading to misreporting due, for example, to desirability bias. Nonetheless, previous validation studies performed on a subsample of the NutriNet-Santé study have supported the good validity of self-reported anthropometric and dietary data [63, 64, 66]. Finally, given that meal planning may be influenced by numerous parameters such as cooking practices and food availability in the surrounding, it is possible that some factors mediating the associations observed in the present paper were not taken into account in the analyses. The potential impact of cooking practices was however considered by adding cooking frequency as a confounder. Future research should be conducted to address the issue of how food availability could potentially influence the relationship observed between meal planning and diet quality.

## Conclusions

Our results highlighted that individuals planning their meals were more likely to have a better dietary quality, including a higher adherence with nutritional guidelines as well as an increased food variety. Additionally, meal planning was associated with lower odds of being obese in men and women and overweight in women only. Although interventional or prospective research should be conducted in order to infer causality, these data suggest the potential interest of promoting meal planning to improve dietary quality and prevent overweight. Such a tool could partly address the issue of time scarcity reported by consumers for meal preparation and, might therefore encourage home cooking. Given the potential benefits of meal planning identified in this study, it would be interesting that future research evaluate the appropriation and the impact of applications designed to help individuals planning their meals.

## Additional file

Additional file 1: Table S1. French Nutrition and Health Program-Guideline Score (PNNS-GS) computation. (DOCX 16 kb)

## Abbreviations

CU: Consumption Units
FFQ: Food Frequency Questionnaire
mPNNS-GS: Modified Programme National Nutrition Santé-Guideline Score
