# Supplementary material for: Meal planning is associated with food variety, diet quality and body weight status in a large sample of French adults
Source: Int J Behav Nutr Phys Act. 2017 Feb 2;14:12. doi: 10.1186/s12966-017-0461-7 (PMC5288891; doi:10.1186/s12966-017-0461-7)
Supplement: Additional file 1: Table S1. — French Nutrition and Health Program-Guideline Score (PNNS-GS) computation. (DOCX 16 kb) [file 12966_2017_461_MOESM1_ESM.docx]

**Additional file**

**Table S1: French Nutrition and Health Program-Guideline Score (PNNS-GS) computation**

|  | **Recommendation** | **Scoring criteria^1^** | **Score** |
| --- | --- | --- | --- |
| **Fruits and vegetables** | At least 5 per day | [0 – 3.5[ | 0 |
|  |  | [3.5 – 5[ | 0.5 |
|  |  | [5 – 7.5[ | 1 |
|  |  | ≥ 7.5 | 2 |
| **Bread, cereals, potatoes and legumes** | At each meal according to appetite | [0 – 1[ | 0 |
|  |  | [1 – 3[ | 0.5 |
|  |  | [3 – 6[ | 1 |
|  |  | ≥ 6 | 0.5 |
| *Whole grain food* | *Preferentially choose whole grains and whole grain breads* | [0 – 1/3[ | 0 |
|  |  | [1/3 – 2/3[ | 0.5 |
|  |  | ≥ 2/3 | 1 |
| **Milk and dairy products** | 3 per day  (≥ 55 years: 3 to 4 per day) | [0 – 1[ | 0 |
|  |  | [1 – 2.5[ | 0.5 |
|  |  | [2.5 – 3.5] (≥ 55-year-old subjects: [2.5 – 4.5]) | 1 |
|  |  | > 3.5 (≥ 55-year-old subjects: > 4.5) | 0 |
| **Meat and poultry, seafood and eggs** | 1 to 2 per day | 0 | 0 |
|  |  | ]0-1[ | 0.5 |
|  |  | [1 – 2] | 1 |
|  |  | > 2 | 0 |
| *Seafood* | *At least twice a week* | < 2 servings per week | 0 |
|  |  | ≥ 2 servings per week | 1 |
| **Added fats^2^** | Limit consumption | Lipids from added fats> 16%EI^3^ per day | 0 |
|  |  | Lipids from added fats≤ 16%EI^3^ per day | 1 |
| *Vegetable added fats* | *Favor fats of vegetable origin* | No use of vegetable oil or ratio vegetable oil/total added fats ≤0.5 | 0 |
|  |  | No use of added fats or ratio vegetable oil/total added fats >0.5 | 1 |
| **Sweetened foods^2^** | Limit consumption | Added sugars from sweetened foods ≥15%EI^3^ per day | -0.5 |
|  |  | Added sugars from sweetened foods [10 – 15[%EI^3^ per day | 0 |
|  |  | Added sugars from sweetened foods < 10%EI^3^ per day | 1 |
| **Beverages** | | | |
| *Water and soda*^2^ | Drink water as desired  Limit sweetened beverages: no more than one glass per day | < 1 L of water and > 250 mL of soda per day | 0 |
|  |  | ≥ 1 L of water and > 250 mL of soda per day | 0.50 |
|  |  | < 1 L of water and ≤ 250 mL of soda per day | 0.75 |
|  |  | ≥ 1 L of water and ≤ 250 mL of soda per day | 1 |
| *Alcohol* | Women^4^ advised to drink ≤ 2 glasses of wine per day and ≤ 3 glasses per day for men. | Ethanol >20 g/d for women and >30 g for men | 0 |
|  |  | Ethanol ≤20 g/d for women and ≤30 g for men | 0.8 |
|  |  | Abstainers and irregular consumers (< once a week) | 1 |
| **Salt^2^** | Limit consumption | > 12 g /d | -0.5 |
|  |  | ]10 – 12] g /d | 0 |
|  |  | ]8 – 10] g /d | 0.5 |
|  |  | ]6 – 8] g /d | 1 |
|  |  | ≤ 6 g /d | 1.5 |
| **Physical activity** | At least the equivalent of 30 min of brisk walking per day | [0 – 30[min /d | 0 |
|  |  | [30 – 60[min / d | 1 |
|  |  | ≥ 60 min /d | 1.5 |

^1^ Servings per day unless otherwise noted

^2^ Established according to the French RDA

^3^ EI: total energy intake without alcohol

^4^ Pregnant women are advised to abstain from all alcohol consumption during the entire duration of pregnancy
